# Supplementary material for: Causal Relationship between Aging and Anorexia Nervosa: A White-Matter-Microstructure-Mediated Mendelian Randomization Analysis
Source: Biomedicines. 2024 Aug 16;12(8):1874. doi: 10.3390/biomedicines12081874 (PMC11351342; doi:10.3390/biomedicines12081874)
Supplement: Supplementary file 1 [file biomedicines-12-01874-s001.zip › Supplementary Figure/Figure S4/Singe SNP plot of MR analysis for TL to ubm-b-1791.pdf]

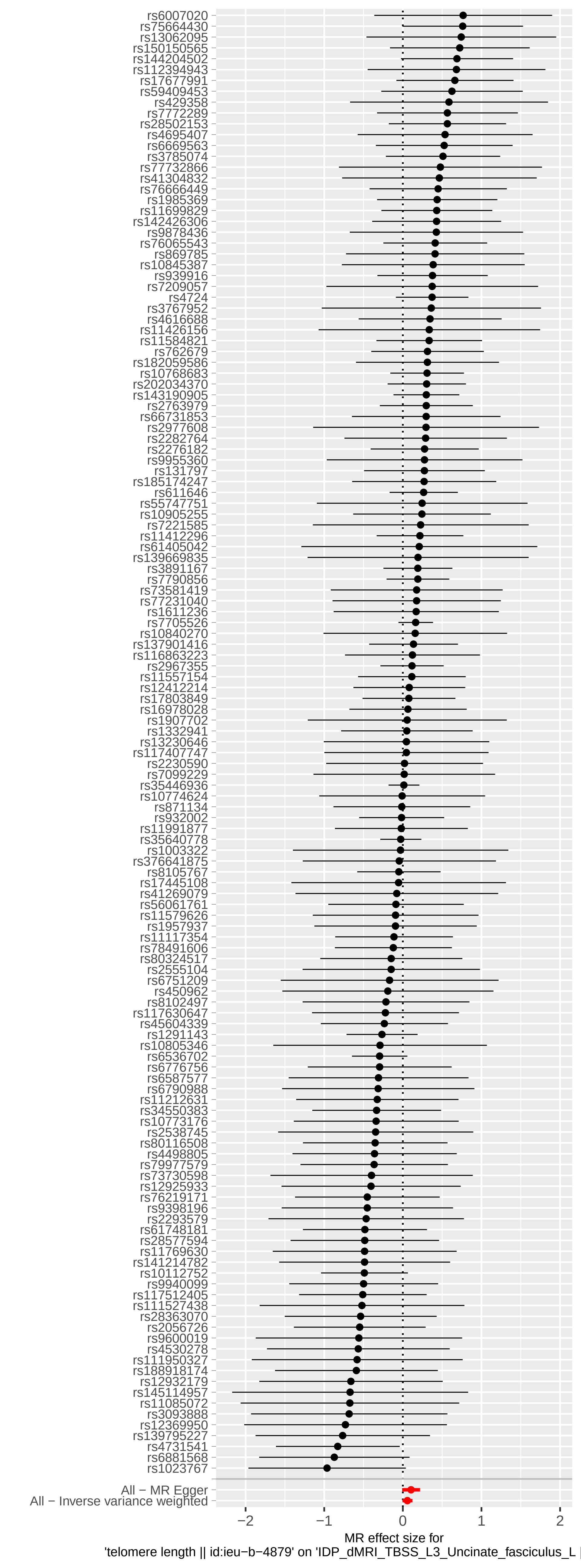
rs6007020
rs75664430
rs13062095
rs150150565
rs144204502
rs112394943
rs17677991
rs59409453
rs429358
rs7772289
rs28502153
rs4695407
rs6669563
rs3785074
rs77732866
rs41304832
rs76666449
rs1985369
rs11699829
rs142426306
rs9878436
rs76065543
rs869785
rs10845387
rs939916
rs7209057
rs4724
rs3767952
rs4616688
rs11426156
rs11584821
rs762679
rs182059586
rs10768683
rs202034370
rs143190905
rs2763979
rs66731853
rs2977608
rs2282764
rs2276182
rs9955360
rs131797
rs185174247
rs611646
rs55747751
rs10905255
rs7221585
rs11412296
rs61405042
rs139669835
rs3891167
rs7790856
rs73581419
rs77231040
rs1611236
rs7705526
rs10840270
rs137901416
rs116863223
rs2967355
rs11557154
rs12412214
rs17803849
rs16978028
rs1907702
rs1332941
rs13230646
rs117407747
rs2230590
rs7099229
rs35446936
rs10774624
rs871134
rs932002
rs11991877
rs35640778
rs1003322
rs376641875
rs8105767
rs17445108
rs41269079
rs56061761
rs11579626
rs1957937
rs11117354
rs78491606
rs80324517
rs2555104
rs6751209
rs450962
rs8102497
rs117630647
rs45604339
rs1291143
rs10805346
rs6536702
rs6776756
rs6587577
rs6790988
rs11212631
rs34550383
rs10773176
rs2538745
rs80116508
rs4498805
rs79977579
rs73730598
rs12925933
rs76219171
rs9398196
rs2293579
rs61748181
rs28577594
rs11769630
rs141214782
rs10112752
rs9940099
rs117512405
rs111527438
rs28363070
rs2056726
rs9600019
rs4530278
rs111950327
rs188918174
rs12932179
rs145114957
rs11085072
rs3093888
rs12369950
rs139795227
rs4731541
rs6881568
rs1023767
All − MR Egger
All − Inverse variance weighted
−2
−1
0
1
2
MR effect size for
'telomere length || id:ieu−b−4879' on 'IDP_dMRI_TBSS_L3_Uncinate_fasciculus_L
